# Tolerogenic Dendritic Cells for Regulatory T Cell Induction in Man

**DOI:** 10.3389/fimmu.2015.00569

**Published:** 2015-11-09

**Authors:** Verena K. Raker, Matthias P. Domogalla, Kerstin Steinbrink

**Affiliations:** ^1^Department of Dermatology, University Medical Center Mainz, Johannes Gutenberg-University Mainz, Mainz, Germany

**Keywords:** tolerance, dendritic cell, regulatory T cells, humans, IL-10, vaccination, study

## Abstract

Dendritic cells (DCs) are highly specialized professional antigen-presenting cells that regulate immune responses, maintaining the balance between tolerance and immunity. Mechanisms via which they can promote central and peripheral tolerance include clonal deletion, the inhibition of memory T cell responses, T cell anergy, and induction of regulatory T cells (Tregs). These properties have led to the analysis of human tolerogenic DCs as a therapeutic strategy for the induction or re-establishment of tolerance. In recent years, numerous protocols for the generation of human tolerogenic DCs have been developed and their tolerogenic mechanisms, including induction of Tregs, are relatively well understood. Phase I trials have been conducted in autoimmune disease, with results that emphasize the feasibility and safety of treatments with tolerogenic DCs. Therefore, the scientific rationale for the use of tolerogenic DCs therapy in the fields of transplantation medicine and allergic and autoimmune diseases is strong. This review will give an overview on efforts and protocols to generate human tolerogenic DCs with focus on IL-10-modulated DCs as inducers of Tregs and discuss their clinical applications and challenges faced in further developing this form of immunotherapy.

## Introduction

Dendritic cells (DCs) have been progressively established as central players in immunity and tolerance. They are the most potent antigen-presenting cell (APC) of the immune system and termed professional APC as a result of their unique ability to capture and to present antigens to T cells, in particular for the stimulation of naïve T cells. This process is characterized as a bidirectional communication between DCs and T cells, and results in the polarization and differentiation of various effector and regulatory T cell subpopulations. It is generally accepted that DCs play pivotal roles in tolerance induction and maintaining of immune-homeostasis and is explained further by their plasticity. DCs implicated in tolerance are in a different state of activation and/or differentiation. The surrounding microenvironment of DCs can affect the activation state of DCs leading to regulatory immune cells but it has also become evident that specialized subsets of DCs promote and maintain tissue homeostasis and tolerance. Focusing on the understanding of the immunobiology of tolerogenic DCs has given us substantial insights on how we can generate and employ tolerogenic DCs for immunotherapeutic applications.

## DCs as Regulators of Immunity and Tolerance

Dendritic cells are a heterogeneous group of APC, which act as highly efficient regulators of immunity and key sentinels in a variety of tissues or lymphoid organs. In this regard, DCs can either function as highly potent and specific inducers of immunity via the activation of lymphocytes and secretion of inflammatory mediators or as inducers of tolerance by various mechanisms of tolerance such as anergy and deletion of T cells or the instruction of Tregs (1).

Immature tissue-resident DCs encounter potential antigens via innate pattern-recognition receptors (PRRs), such as toll-like receptors (TLRs) or c-type-lectin receptors, take up the antigens via micropinocytosis and degrade them into smaller peptides, which can be presented to other immunes cells by surface displayed major histocompatibility complexes (MHC) (2). The antigen uptake triggers maturation processes of DCs that result in the upregulation of costimulatory molecules like CD40, CD80, CD86 and secretion of proinflammatory cytokines/interleukines (IL) like IL-1β, IL-12, IL-6, and TNF as well as more MHC–peptide complexes (3). In order to reach T cells in secondary lymphoid organs, DCs upregulate trafficking receptors, such as CCR7, which enable them for migration to lymph nodes wherein they encounter naïve T cells which recognize the MHC–peptide complex with an antigen-specific T cell receptor (4). Sufficient activation and antigen recognition subsequently activate T cells to differentiate into T helper cells or cytotoxic T effector cells. With this in mind, it is easy to appreciate that DCs function as an important link between innate and adaptive immune responses.

Apart from the induction of efficient immune responses against invading pathogens, DCs do also exhibit regulatory functions in order to maintain central and peripheral tolerance. During steady state, DCs capture self-antigens and silence auto-reactive T cells (5). So-called tolerogenic DCs bear low amounts of costimulatory molecules on their surface and exhibit reduced secretion of proinflammatory IL-12 but high production of anti-inflammatory cytokines like IL-10 (6, 7). Tolerogenic DCs provide insufficient stimulatory signals for T cells and therefore drive naïve T cells to differentiate into Tregs rather than T effector cells (8). DCs which are not activated after phagocytosis of, for example apoptotic cells, exhibit a tolerogenic function via the secretion of transforming-growth-factor-beta (TGF-β) and subsequent induction of Foxp3^+^ Tregs in the draining lymph nodes (9). Tregs can be induced by a variety of DCs *in vivo*. For one CD103^+^ DCs, which in the presence of retinoic acid in the gut, are able to activate gut T cell homing via CCR9 and α4β7 (10, 11). TGF-β and retinoic acid enhance the number of Foxp3^+^ Tregs, which is an example of how tolerogenic DCs in the gut induce Tregs (12, 13).

Although a variety of TLRs, such as TLR3, TLR4, TLR5, TLR7, and TLR8, can efficiently induce immune responses via DCs, TLR2, for instance, facilitates tolerance induction via promotion of IL-10 producing tolerogenic DCs *in vivo* inhibiting type I interferon production via an inhibition of the TLR7/9 signaling pathway (14, 15). The maturation state of DCs alone does not define their potential to induce Tregs. In addition, the nature of the pattern recognition receptors or the expression of costimulatory or coinhibitory molecules by DCs affects the resulting immune response as well. Fully matured DCs are sufficient in the induction of T helper cell differentiation. Incomplete maturation of DCs (semi-mature DCs) or expression of inhibitory surface molecules results in the activation of Tregs, e.g., IL-10 producing T cells with regulatory potential in experimental autoimmune encephalomyelitis (EAE) (16, 17).

## Mechanisms of Induction and Function of Tolerogenic DCs

When analyzing tumor escape mechanisms scientists observed that cancer cells and the associated stroma converted myeloid DCs in the tumor microenvironment into tolerogenic phenotypes in order to induce Tregs, which subsequently dampened anti-tumor immunity (18, 19). The pool of tolerogenic and regulatory DCs is very heterogeneous and can be divided in naturally occurring regulatory DCs and induced tolerogenic DCs (5). Thymic DCs contribute to central tolerance induction by presentation of self-antigen to thymocytes and are most likely influenced by thymic stromal lymphopoetin (TSLP) to show a tolerogenic phenotype and function (20). Most of the DCs described in certain tissues like pulmonary plasmacytoid or myeloid DCs have tolerogenic functions under steady state conditions.

Immature DCs (iDCs) are poorly immunogenic because of low surface expression of costimulatory molecules and only modest MHCII levels. Therefore, iDCs themselves are tolerance inducers under steady state conditions. Furthermore, repetitive stimulation of T cells with human iDCs can convert naïve T cells to Tregs (21, 22). This was also addressed in murine studies where antigen was given to mice without further maturation signals. Antigen-loaded DCs accumulated in secondary lymphoid organs where they promoted Treg differentiation and proliferation rather than inducing T effector cells (23).

In mucosal tissues such as lung and gut where a constant exposure to a variety of foreign antigens is given, DCs are kept in a tolerance promoting state by the action of IL-10 and TGF-β or enhanced production of CCL18 in the surrounding micro-milieu (4, 24, 25). Most of these tolerogenic occurrences can be overwritten by inflammatory signals that convert tolerogenic DCs into an inflammatory phenotype. Though this is not the case for Langerhans cells (LCs) found in human skin as they most likely lack a high expression of PRRs like TLRs (5) and have been associated with tolerance induction as well as immunity. During leishmaniasis, parasite-infected DCs mediate protection against the infection by IL-12 production (26), but it has also been shown that a selective depletion of LCs from the DC population in the skin can attenuate the disease accompanied by increased numbers of CD4^+^Foxp3^+^ Tregs (27). In contact hypersensitivity (CHS) models, the role of LCs has also been controversially discussed. When UVR-depletion of LCs occurs during the sensitization phase, the ear swelling responses in CHS are reduced and Tregs are induced, but this is largely depending on the area and time of depletion (28, 29). Tolerogenic functions of LCs are mainly based on their low migratory properties, low expression of costimulatory molecules, and low secretion of cytokines (30).

Besides delivering costimulatory signals to T cells DCs also function as producers of mediators such as IL-12, a proinflammatory cytokine driving Th1 cell differentiation of naïve T cells, or tolerance-promoting IL-10 on the other hand (31–33). Interleukin 10 produced by tolerogenic iDCs is a prerequisite for Treg induction in a variety of different tolerance models like allergy and autoimmunity (33, 34). Other factors secreted by tolerogenic DCs involve TGF-β, although it is not clear if the tolerogenic capacity of DCs relies on TGF-β production because TGF-β can promote Treg differentiation and drive Foxp3 expression in Tregs in the absence of DCs as well (5).

In mice that have been exposed to low doses of a contact allergen, the cross talk between DCs and Tregs plays an important role to induce a protective mechanism against contact hypersensitivity (CHS) reactions (35). During low-zone tolerance CD4^+^CD25^+^ Tregs contact CD11c^+^ DCs via gap junctions and render them tolerogenic resulting in subsequent induction of contact allergen-specific Tregs which inhibit the action of CD8^+^ T effector cells in CHS (35). In the human system, cell aggregates of Tregs and DCs have been observed pointing toward a DC/Treg cross talk inhibiting the maturation process in DCs (36).

Notably different populations of Tregs require various levels of costimulation provided by DCs. A strong CD80/CD86 signal may be sufficient in maintaining thymus-derived Tregs but low or no costimulation is required for maintenance Foxp3^+^ Tregs (37). Besides costimulatory molecules, DCs do also display membrane receptors that may modulate T effector cells during activation, in particular, immunoglobuline-like transcripts (ILT) receptors. This context, ILT4 is exclusively expressed on human myelomonocytic cells and interacts with MHCI molecules on T cells inhibiting subsequent activation (38–40). Programed death ligand 1 (PD-L1) and PD-L2 are expressed by human DCs activating T cells by engagement of T cell displayed PD-1 (41). Upregulation of PD-1 occurs after repetitive stimulations of T cells (e.g., in chronic viral infections) and is a characteristic of “exhausted” T cells. Effects facilitated by PD-1 resemble in most parts IL-10 receptor (IL-10R) pathways such as limitation of PI3K activation and restriction of costimulatory signaling (42).

Another mechanism by which DCs, in particular plasmacytoid DCs (pDCs), indirectly drive Treg differentiation is mediated by indoleamin-2,3-dioxygenase (IDO). The IDO-dependent degradation of the essential amino acid tryptophan around T cells and concurrent production of toxic metabolites like kynurenines leads to an inhibition of translation efficiency and apoptosis induction in T cells (43). Apart from pDCs, IDO was also found on myeloid DCs in chronic hepatitis C infection in which IDO inhibitors may function as potent treatment options for patients (44).

The broad spectrum of tolerogenic DCs and the DC-induced Treg/T effector responses, which arise in the immune system are a challenge to clinical concepts in the treatment of allergies, autoimmunity, and allograft rejections and have to be critically discussed when DCs are modulated (tolerized) *in vitro* for clinical applications.

## Induced Tolerogenic DCs in Man

**Figure 1:**
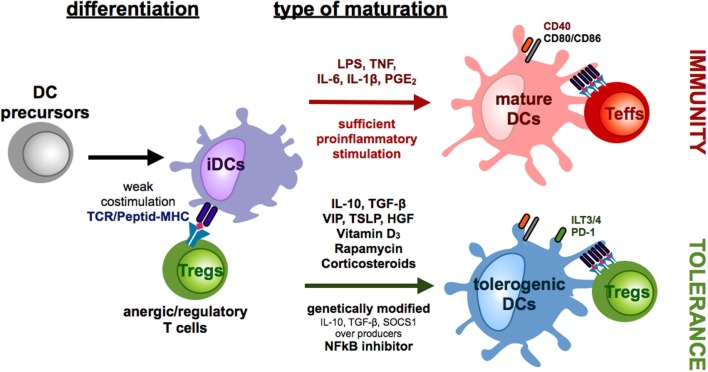
**Differentiation of monocyte-derived tolerogenic DCs**. DCs differentiate from DC precursors in the peripheral blood under the influence of IL-4 and GM-CSF *in vitro* into immature DCs (iDCs). Repetitive stimulations of T cells with iDCs result in the induction of anergic/regulatory T cells (Tregs). In the presence of sufficient maturation signals, which are provided by bacterial components via toll-like receptors or by distinct combinations of proinflammatory cytokines, DCs mature into a migratory/stimulatory phenotype. Incubation of iDCs with several mediators or genetic modification of DCs in the presence or absence of maturation factors can lead to the generation of tolerogenic DCs, which inhibit effector/cytotoxic T cells responses by induction of anergy, apoptosis, or Tregs.

Immature dendritic cells (iDCs) that ensure immune tolerance under steady state conditions display all properties of tolerogenic DCs cells and it has already been shown that they are capable of inducing tolerance *in vitro* and *in vivo*. However, the main obstacle for application of iDCs for treatment of excessive immune responses is their relatively unstable phenotype under inflammatory conditions (45, 46). The proinflammatory environment of inflammatory disorders would probably lead to activation and maturation of iDCs resulting in immune activation opposing the requested immune tolerance. Furthermore, iDCs exhibit low expression of lymph node homing receptors, so it is very unlikely for them to encounter T cells, which is essential for their function. Therefore, many different protocols for *ex vivo* generation of tolerogenic DCs cells that bear a stabilized phenotype have been established (Figure 1). In general, tolerogenic DCs with individual varying immune-modulatory actions can be induced *in vitro* by a multitude of diverse approaches that include genetic engineering, exposure to immune-modulating pharmacological agents or addition of distinct cytokines and growth factors (47) (Figure 1). As the focus of this review is the induction of Tregs by tolerogenic DCs, it is essential to point out which different populations of Tregs can be generated by tolerogenic DCs *in vitro* and *in vivo*. Several subpopulations of Tregs that differ in the expression of surface markers and their way of function have been identified from which the most important ones are CD4^+^ Foxp3^+^ Tregs and CD4^+^ IL-10 producing Tr1 cells, which both can be induced or activated by tolerogenic DCs (48). Foxp3^+^ Tregs are highly CD25 positive (49) and express low levels of CD127 (50) and of CD49d (51). Furthermore, they mainly exhibit their suppressive capacity via cell-to cell contacts. In contrast, Tr1 Tregs are characterized by high secretion of IL-10 and TGF-β and predominantly induce tolerance by cytokine-mediated mechanisms (52). However, it is worth noting that many Tregs that are induced by tolerogenic DCs are not further characterized regarding surface marker expression and mode of suppression.

Progress in the field of genetic manipulation has raised the opportunity to genetically modify DCs to induce a tolerogenic phenotype and function. For instance, knock-down of proinflammatory cytokines or molecules, such as IL-12 (53) or NFκB (54), leads to a reduced DC maturation and inhibits efficient T cell activation, whereas overexpression of death receptor ligands like, for example, Fas ligand (55) and PD-L1 (56) or immunoregulatory proteins like IDO (57) and cytotoxic T lymphocyte antigen 4 (58) directly induce apoptosis or suppress the immunogenic function of the responding T cells (Figure 1). However, genetically engineered tolerogenic DCs can also be used to induce Tregs as DCs with genetically enhanced IL-10, TGF-β, or SOCS1 expression sustain an immature phenotype and promote the induction of Tregs (59–61) (Figure 1).

**Figure 2:**
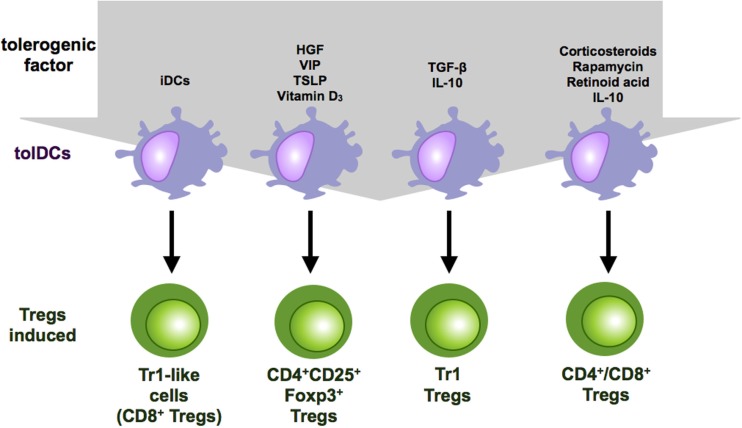
**Regulatory T cell subsets induced by tolerogenic DCs**. Tolerogenic DCs can induce a variety of Treg populations depending on the culture conditions and the tolerogenic factor they encountered during differentiation. Repetitive stimulation of T cells with iDCs leads to anergy or Treg induction. IL-10-modulated DCs induce Tr-1 cells or CD4^+^ and CD8^+^ suppressor T cells. Vitamin D3 and corticosteroids favor the induction of CD4^+^CD25^+^Foxp3^+^ Tregs.

Furthermore, tolerogenic DCs can be induced by various different immune-modulating pharmacological agents that, among others, are vitamin D3, corticosteroids, rapamycin, cyclosporine, tacrolimus, aspirin, and retinoid acid (5) (Figure 1). Depending on the length and time point of drug treatment, some of these are also capable of inducing Tregs (Figure 2). First, exposure of vitamin D3 to monocyte-derived DCs results in the induction of semi-mature DCs, characterized by low expression of costimulatory molecules but augmented levels of IDO, IL-10, TNF-related apoptosis-induced ligand (Trail) and PDL-1 in which the latter ones are critical for the induction of IL-10 expressing Tregs (41, 62, 63). Intriguingly, vitamin D3 exposed to different DC subsets leads to induction of distinct Treg populations. Addition of vitamin D3 to skin LCs or CD141^−^CD1c^+^ blood cells results in induction of CD25^+^Foxp3^+^ Tregs (64, 65) whereas exposure of dermal DCs to vitamin D3 results in Foxp3^−^ Tr1 cells (65). Corticosteroids are well known as immunosuppressive agents and frequently used as drugs in transplantation medicine. The immunosuppressive capacity of corticosteroids at least partially depends on the induction of tolerogenic DCs (66). For example, dexamethasone-induced tolerogenic DCs that are characterized by low expression of MHC II and costimulatory molecules and enhanced levels of ILT2, ILT3 expression, and IL-10 secretion acquire the ability to induce contact-dependent Tregs that suppress T cell responses in an antigen-dependent manner (41). Additionally, treatment with prednisolone induces tolerogenic DCs that provoke regulatory T cell induction (67). Another immunosuppressive drug, rapamycin, that inhibits the protein kinase mTOR (mammalian target of rapamycin) has also been reported to induce CD4^+^CD25^+^Foxp3^+^ T cells either by acting directly on T cell differentiation (68) or indirectly via induction of tolerogenic DCs that stimulate Treg expansion *in vitro* and *in vivo* (69–71) (Figure 2). Moreover, as already mentioned above, reduced NFκB activation in DCs leads to induction of a tolerogenic phenotype. In line with these results, treatment of DCs with the NFκB inhibitor BAY-117085 elicits Treg-inducing capabilities (72).

In addition to genetic manipulation and immune-modulatory drugs, different cytokines and growth factors have also been reported to induce tolerogenic DCs. These include hepatocyte growth factor (HGF), vascular intestinal peptide (VIP), thymic stromal lymphopoietin, PGE_2_, GM-CSF, and TNF, some of which can also induce tolerogenic DCs with Treg inductive capacity (73) (Figures 1 and 2). DCs, generated in the presence of HGF, display a tolerogenic phenotype characterized by a high IL-10/IL-12 ratio and are capable of inducing Foxp3^+^ Tregs that show high expression of IL-10 in an at least partially ILT-3 dependent manner (74). Furthermore, among other immunosuppressive functions, the immune-modulatory neuropeptide VIP has also been reported to induce tolerogenic DCs that are potent inducers of regulatory Tr1 cells that secrete high levels of IL-10 and TGF-β (75, 76). However, there are also different reports that VIP-treated DCs induce CD4^+^CD25^+^Foxp3^+^ Tregs (77–79) (Figure 2). Additionally, thymic stromal lymphoprotein enables DCs to induce differentiation and proliferation of CD4^+^CD25^+^Foxp3^+^ T cells from CD4^+^CD25^−^ thymocytes (80).

Last but not least, the two most prominent immunosuppressive cytokines, TGF-β and IL-10, alone or in combination are also capable of inducing tolerogenic DCs that acquire the ability to induce Tregs. However, as a study by Boks et al. identified IL-10-modulated DCs as the most potent tolerogenic DC subset for clinical application, they will be further highlighted in the next section (81).

## IL-10-Modulated Tolerogenic DCs as Inducers of Regulatory T Cells

The immune-modulatory cytokine IL-10 plays an indispensable role in the induction of tolerance and the limitation of excessive immune responses. IL-10 exerts its immunosuppressive function after binding to the IL-10 receptor complex that further leads to downstream activation and homodimerization of STAT3 through tyrosin phosphorylation of Tyk2/JAK1 (82). In general, IL-10 acts on DCs by downregulation of MHC II and costimulatory molecules in combination with reduced release of IL-6, IL-1β, TNF, and most prominent IL-12 (83–86) Additionally, DCs that are cultured in the presence of IL-10 upregulate the expression of inhibitory molecules like HLA-G (85, 87, 88), ILT2, ILT4 (89), and HO-1 (90) and produce more IL-10 (91), resulting in a positive feedback loop.

**Figure 3:**
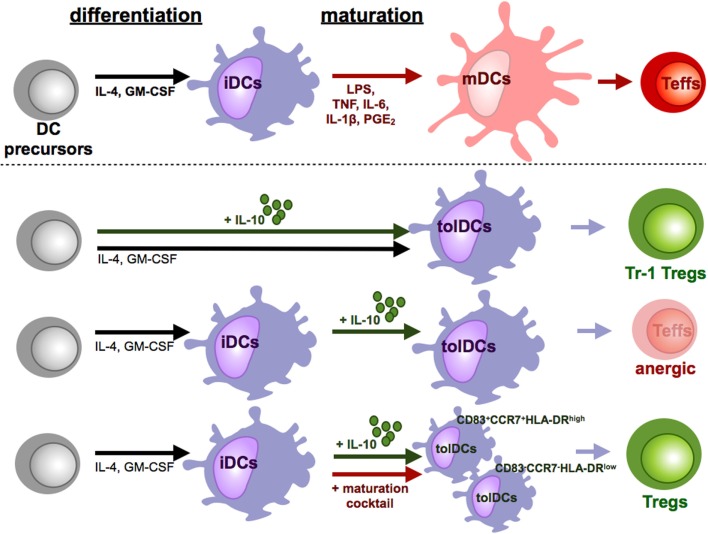
**Tolerizing effects of IL-10 during DC culture**. Under influence of proinflammatory cytokines and other soluble mediators and pattern recognition receptor ligands iDCs differentiate into mDCs. In contrast to these fully mature DCs, IL-10 induced various subpopulations of tolerogenic DCs (tolDCs). The time point and duration of IL-10 supplementation is crucial for the induction of tolDCs. When IL-10 is present during the whole culture, tolDCs favor the induction of Tr-1 like cells. In cultures of iDCs with IL-10, which did not undergo maturation, tolDCs are generated which induce T cell anergy or deletion of T cells. Addition of IL-10 only during the maturation step differentiates DCs to a stable tolerogenic subpopulation that induces suppressive CD4^+^ and CD8^+^ Tregs.

Many different protocols for the addition of exogenous IL-10 to DC cultures have been established (Figure 3). Addition of IL-10 during the entire DC culture starting with monocytes in the presence of IL-4 and GM-CSF results in cells that exhibit a macrophage-like phenotype characterized by high expression of CD14 and CD16. Therefore, it was assumed that IL-10, during early stages, inhibits DC generation by favoring macrophage differentiation (92). However, it has subsequently been reported that IL-10 added at early stages of DC generation out of monocytes in the presence of IL-4 and GM-CSF results in CD14^+^CD16^+^CD83^+^CD86^+^ and HLA-DR^+^ tolerogenic myeloid cells that additionally express tolerance-associated proteins like HLA-G, ILT2, ILT3, and ILT4 and secrete high amounts of IL-10 (88). These IL-10-modulated DCs (DC-10) are phenotypically and functionally stable after stimulation. Moreover, they are capable of inducing antigen-specific, IL-10 producing Tr1 Tregs in an ILT4-, HLA-G-, and IL-10-dependent mechanism (85, 88) (Figure 3). In mixed leukocyte reaction, those induced Tr1 Tregs that can be characterized by coexpression of CD49b and LAG-3 suppress primary T cell responses by secretion of IL-10 and TGF-β (93). Although, when IL-10 is added after generation of iDCs without additional maturation stimuli, the resulting DCs express the tolerogenic IL-10 DCs phenotype that is described above and acquire the ability to induce anergic T cells though no regulatory activity of those anergic T cells was detected (94) (Figure 3). Intriguingly, simultaneous addition of IL-10 to a strong maturation stimulus that consists of IL-6, IL1β, PGE_2_, and TNF results in DCs with even stronger tolerogenic properties and a stable phenotype under proinflammatory conditions (95, 96). Treatment of naïve CD4^+^ or CD8^+^ T cells with those IL-10-modulated DCs results in induction of anergic Tregs that on their part acquire the capability to inhibit CD4^+^ and CD8^+^ T cell activation and function (Figure 3). In this case, the induction of Tregs is IL-10 and TGF-β independent and at least partially mediated by CTLA-4 (97). Analysis of signal transduction events of the induced Tregs demonstrated downregulation of MAPKs, JNK, and ERK but a significant upregulation of p38. The elevated induction of p38 is essential for expression of the cell cycle inhibitor p27^Kip1^ and required for their suppressive activity (98). Intriguingly, yet unpublished data indicate that those DCs, when IL-10 is added during a maturation step for the last two days of DC culture, are consisting of two distinct subpopulations: an immature phenotype of CCR7^−^CD83^−^HLA-DR^low^ and a mature subset of CCR7^+^CD83^+^HLA-DR^high^ IL-10 DCs but both express co-inhibitory molecules ILT-3 and ILT-4 (Kryczanowsky and Steinbrink, unpublished observations) (Figure 3). However, the CCR7^+^CD83^+^HLA-DR^high^ IL-10 DC subpopulation induces a Treg population with a stronger suppressive capacity, exerts high migratory capacity toward secondary lymphoid organs, and displays a stable phenotype under inflammatory conditions. When IL-10 is added to fully mature DCs, it has no tolerogenic effect on DC function due to down-regulated IL-10 receptor expression (95, 99).

Furthermore, Gregori et al. demonstrated the existence of IL-10-modulated DCs in man *in vivo*. Those represent 0.3% of the blood mononuclear cells and differ from mDCs and pDCs as they are CD14^bright^, CD16^+^ and express CD83 (88). They can also be separated by macrophages due to their DC morphology. Furthermore, they are CD11c^+^, CD11b^+^ and express CD80, CD86, and HLA-DR. (88). This suggested important role for IL-10-modulated DCs *in vivo* is further emphasized by patients that suffer from hyper IgE syndrome, a disease that results from deficient IL-10 signaling due to defective STAT3. DCs isolated from those patients show an impaired sensitivity toward IL-10 leading to a reduced capacity to induce Tregs (100).

Comparative analysis of a multitude of protocols of monocyte-derived tolerogenic DCs demonstrated that Tregs induced by IL10-DCs display an enhanced suppressive capacity compared to Tregs generated by TGF-β-, dexamethasone-, vitamin D3-, or rapamycin-induced tolerogenic DCs (81). Moreover, IL-10-modulated DCs are terminally differentiated as they are stable under various different inflammatory conditions what makes them a promising tool for *in vivo* application in diseases that are linked to enhance immune activation.

## Clinical Application of Tolerogenic DCs

The data discussed above greatly expand our current understanding of the plasticity of distinct tolerogenic DC subsets in regulation of inflammation and homeostasis. The most challenging issue is now to translate our knowledge of tolerogenic DCs into preclinical mouse models and into patients to prove the therapeutic potential of DCs in man.

In contrast to tolerogenic DCs, immunostimulatory DCs are increasingly utilized in cancer immunotherapy (101, 102). The vast majority of these DC vaccination trials in cancer revealed that the administration of autologous DCs is well tolerable and strongly immunogenic and multiple approaches for DC generation, maturation, antigen loading, and application routes and doses have been developed and tested, so that these comprehensive knowledge may support the development of DC vaccination strategies with tolerogenic DCs. Specific therapy to prevent or to inhibit immune activation is highly desired in allergic and autoimmune diseases and in transplantation medicine. Current therapies, which include immunosuppressive drugs, often do not specifically target the cause of disease or transplant rejection and can be associated with severe side effects. *Ex vivo* generated tolerogenic DCs are therefore an attractive preventive or therapeutic approach to enhance, maintain or restore immunological tolerance. Evidence from a multitude of animal models strongly demonstrated the efficiency of tolerogenic DCs in the fields of allergy, autoimmunity and transplantation medicine (5, 103). However, for DC-based immunotherapy in man, the methods for tolerogenic DC generation have to be converted into clinically applicable protocols and the properties of tolerogenic DCs, including phenotype, stability, migratory capacity, and mode of tolerance induction (e.g., T cell anergy or apoptosis, induction of Tregs, interaction with other immune cells) have to be analyzed in comparative studies.

There have been a large number of *in vitro* studies performed as a proof-of-principle that human tolerogenic DCs can efficiently reduce effector T cell responses, in part by activation or expansion of Tregs as discussed above (25, 64, 66, 68, 81, 100). In this context, it has been shown that DCs can act in an antigen-specific fashion after loading with endogenous or exogenous antigens. Excitingly, several groups have started to study the properties of tolerogenic DCs in patients suffering from allergic or autoimmune diseases and to analyze the potential of tolerogenic DCs in treatment of allergic, inflammatory and autoimmune disorders. Vitamin D3-conditioned tolerogenic DCs obtained from relapsing–remitting multiple sclerosis patients loaded with myelin peptides as specific antigen, expressed a semi-mature phenotype and an anti-inflammatory profile and induced a stable antigen-specific T cell hypo-responsiveness (66). Another study revealed that clinically grade dexamethasone and vitamin D3-treated tolerogenic DCs from patients with rheumatoid arthritis suppressed mature antigen-specific DCs induced T cell activation and rendered T cells unresponsive to further restimulation (104). In addition, IL-10-modulated DCs generated from atopic asthmatic donors suppress specific allergen-driven proliferative and Th2 responses of autologous effector T cells and convert these effector T cells into Tregs (105).

One of the major concerns associated with tolerogenic DC-based immunotherapy is the functional stability of the regulatory phenotype, particularly when targeting inflammatory diseases (e.g., allergies and autoimmune diseases). DCs express receptors for chemokines, growth factors, and PRRs that can be activated by a number of proinflammatory mediators and microbial and non-microbial agents in the microenvironment (106–108). Therefore, a potential risk of *ex vivo*-generated tolerogenic DCs is that they lose their regulatory properties and switch to an immunostimulatory phenotype, resulting in an activation rather inhibition of (antigen-specific) immune responses. Thus, clinically applicable tolerogenic DCs must be tested rigorously for robust stability before transfusion into patients to assess the impact of maturation provoking or otherwise inflammatory signals on the tolerogenic phenotype of differentiated DCs. In this context, Boks et al. performed a comparative analysis of several *ex vivo* generated tolerogenic DCs to test their potential for clinical applications as discussed above (81). Among other characteristics, they found that IL-10-modulated tolerogenic DCs maintained their regulatory properties in the presence of TLR and proinflammatory cytokines whereas dexamethasone-, rapamycin-, or TGF-β-induced DCs in part lost their tolerogenic phenotype and their capacity of immune regulation. Furthermore, important for the induction of suppressive immune responses in T cells is the CCR7-directed migratory capacity of tolerogenic DCs toward the secondary lymphatic organs (109). Therefore, a high and under proinflammatory condition stable expression of CCR7, resulting in a high migratory capability, as shown for IL-10-modulated DCs (Kryczanowsky and Steinbrink, unpublished observation), is a prerequisite for an optimal tolerogenic DCs subset as candidate for *in vivo* vaccination studies. In addition, the route, dose, and frequency of DC application have to be identified in man for the development of optimized protocol for tolerogenic DC-based immunotherapy. In a mouse model of collagen-induced arthritis, low doses of DCs showed excellent anti-arthritis activity by induction of Foxp3^+^ Tregs, whereas high numbers accelerated arthritis symptoms (45). In contrast to these results, other groups using high number of tolerogenic DCs found a protective effect (110–112). These different results may be due to different routes of applications used. In the studies with high doses, tolerogenic DCs were administered by the i.v. or i.p. route whereas in the study where low dose application proved to be more effective, the DCs were injected s.c. Therefore, further studies addressing tolerogenic DCs migration *in vivo* will be useful to determine the optimal route, dose, and frequency of application for each tolerogenic DC subset and disease.

The first study of tolerogenic DCs in man was conducted in 2001 in Ralf Steinman’s lab. They used iDCs, generated in the presence of IL-4 and GM-CSF which were pulsed with antigens and subsequently s.c. injected (2 × 10^7^/subject) into healthy donors (113, 114). They demonstrated that the DC administration was well tolerated and that the treatment suppressed antigen-specific CD8^+^ T cells responses and that this immune regulation lasted for >6 months. They were the first to show the tolerogenic effect of DCs *in vivo* and at this time their results urge caution with the use of iDCs for enhancement of tumor or microbial immunity.

More recently, a randomized, double-blind phase I study was conducted in type I diabetic patients who required insulin treatment for at least 5 years (115). Patients were injected with autologous monocyte-derived DCs that were either un-manipulated (control) or were treated *ex vivo* with anti-sense oligonucleotides targeting CD40, CD80, and CD86 to silence these molecules. During the trial protocol, the 10 study patients were i.d. injected with 1 × 10^7^ DCs four times at 2-week intervals and were monitored subsequently for a period of 12 months. DC treatment was well tolerated without any side effects and did not induce autoantibody production. In addition, patients did not lose their capability to mount T cell responses to viral or allogeneic cells, indicating the absence of systemic immunosuppression. Analysis of the immune response *in vivo* after vaccination with these DCs revealed no alteration in the composition and activation of immune cells/responses with exception of increased IL-4 and IL-10 levels and an upregulation of the frequency of potentially beneficial B220^+^CD11c^−^ B cell population whose suppressive activity was shown in *in vitro* experiments. Overall, there were no significant differences between control and tolerogenic DCs in all parameters tested.

Another clinical phase I trial was conducted in patients suffering from rheumatoid arthritis. Here, tolerogenic DCs were generated in the presence of an NF-κB inhibitor. They are deficient for CD40 expression but express CD86 (116) and were loaded with four citrullinated peptide antigens. A total of 18 patients received a single dose (either one or five million) of i.d. applicated tolerogenic DCs and were evaluated at baseline, and after 3 and 6 months after therapy. Vaccinations with these tolerogenic DCs were well tolerated and no adverse effects in any patient throughout this study have been observed (117). The main conclusion of these first two trails of tolerogenic DC-based immunotherapy is that i.d. injection of tolerogenic DCs appears to be safe and do not enhance autoimmune responses. Currently, a randomized placebo-controlled dose escalation phase I study was started and is still ongoing in which rheumatoid arthritis patients are injected intra-articularly (AUTODECRA trial) with autologous tolerogenic and antigen-pulsed DCs (117).

## Conclusion

It has been shown that multiple mediators can induce a tolerogenic phenotype in human DCs that, among other mechanisms, exploit their regulatory capacity through the induction or expansion of Tregs. These tolerogenic DCs not only employ secreted mediators and inhibitory receptors to drive Treg generation but can also provide additional signals to direct Tregs to the anatomical site of function.

A better understanding of the phenotypical properties, the precise molecular mechanisms, and the immunological functions of tolerogenic DCs will provide essential information for rational design of tolerogenic DC-based immunotherapies. A major challenge in the future will be to identify the most appropriate tolerogenic DC population for defined applications and to optimize the generation protocols, including dose, route, and frequency of administration. In the long run, these findings will support the development of novel and innovative immunotherapeutic approaches for the control of allergic and autoimmune diseases and allograft rejections.

## Authors Contribution

The authors (VR, MD, and KS) wrote the article.

## Conflict of Interest Statement

The authors declare that the research was conducted in the absence of any commercial or financial relationships that could be construed as a potential conflict of interest.
